# On Catarrh of the Uterus*From his work, “Der Katarrh der Inneren Weiblichen Geschlechtstheile.” Leipzig, 1870. Chap. iii., part ii. Translated by John C. Jay, Jr., M.D., New York.

**Published:** 1872-08

**Authors:** Carl Hennig

**Affiliations:** Professor in the University of Leipsic, formerly Assistant to the Obstetrical Clinique


*On Catarrh of the Uterus.** By Carl Hennig, M.D., Professor in the University of Leipsic, formerly Assistant to the Obstetrical Clinique.

### I. CATARRH OF THE NON-PREGNANT UTERUS.

### A.—PRIOR TO THE MENSTRUAL EPOCHS.

*Pathological Anatomy:* (a) *In Children.*—Catarrh affects the uterus more frequently than the fallopian tubes, and occurs as frequently before the seventh year as afterwards. Before the seventh year, it generally only affects the cavity of the body, and, as a rule in children, never the cervical canal independently, but always the cavity of uterus and cervix simultaneously; then, also, as a rule, the vaginal mucous membrane participates in the catarrh. Only in one of my cases was the peritoneal covering of the catarrhal uterus thickened, in one other the muscular tissue of the anterior uterine wall was hyperæmic, and in a third pale. In general, it was proved by my examinations (differing from the assertion of Aran) that catarrhal affection of the mucous membrane does not, in the majority of cases, cause hyperæmia or inflammation of the uterine muscular tissue, nor should it be considered a result of the latter.

The mucous membrane in this disease has more rugæ than is seen in the healthy uterus during childhood; is not thickened, is of a yellowish or pale orange color, while here and there it is very pale, grayish, and very seldom injected (at least in the cadaver). Acute catarrh I have seen once in a little girl of nine months. The mucous membrane had many folds, was covered with an abundant, perfectly transparent, slightly acid mucus, of the consistency of the white of an egg. This mucus became cloudy upon the addition of diluted acetic acid, and contained, excepting a few pale granular cells, no elements.

In chronic catarrh it is difficult to recognize the tubular glands of the mucous membrane. After the addition of acetic acid, in their interior can be seen pale, round, or obtuse, irregularly arranged, single nucleated cells. The mucus has a faintly acid or slightly alkaline reaction, is transparent, slightly grayish, mucilaginous, and rarely limpid. Under the microscope, within it can be seen numerous cylindrical cells, oftentimes longitudinally striated,

* From his work, "Der Katarrh der Inneren Weiblichen Geschlechtstheile." Leipzig, 1870. Chap. iii., part ii. Translated by John C. Jay, Jr., M.D., New York.

and also a large amount of free hyaline. I never observed any ciliated epithelium, and only rarely blood corpuscles and pigment. Granular cells also are only occasionally met with.

(b) *In adults.*—Uterine catarrh occurs strikingly seldom during the middle of life, but frequently toward the menopause. We find that its occurrence in the cervical canal is very evenly distributed through the different periods of adult life, and that generally catarrh of the cavity of the body of the uterus occurs independently of catarrh of the cervix. Only in old age do the two affections seem to run parallel.

The uterine muscular tissue in catarrh is frequently softer, oftentimes hyperæmic, rarely injected or œdematous, and is in one-ninth of the cases pale and grayish-yellow. The post-mortem thickness and roughnesses of the peritoneal covering are often due to some other cause than the catarrh.

In acute catarrh the mucous membrane is a little swollen, and in young women greatly injected; in more advanced life it is pale. The cavity of the uterus is moderately enlarged, and contains as much as one scruple of glary, not very viscid, slighly yellowish mucus, having a slightly acid reaction, and in old women a strongly alkaline reaction. In it is seen here and there ciliated epithelium.

In chronic catarrh, the mucous membrane is perceptibly thickened only in old women, though occasionally even in young persons it is strongly pigmented, softened, roughened, and pitted, and quite often highly injected. In old women it is sometimes apoplectically hemorrhagic—a condition I have only seen in cholera corpses.

In others, especially after long-continued or oft-repeated catarrhal disturbance, the mucous membrane has a mottled grayish-green or brownish-red (inflammatory) appearance, or is exceedingly pale, with here and there networks of little vessels. Only in old women are the swollen mucous follicles appreciable to the naked eye; they are then lined with cells in a state of fatty or colloid degeneration. In younger women I found only the epithelium swollen and becoming hyaloid, surrounded by granular matter, and only in one woman of fifty-four years were the central cells fatty. In the latter case catarrh was not present.

In old age, cystic degenerated uterine follicles are seldom absent; they originate through a closure of their orifices, whereupon an accumulation of their contents takes place, and the fundus of the gland, therefore, distends into a sack. Accompanying this condition there is always an excess of mucus upon the free surface of the mucous membrane, while here and there can be seen tufts or glandular polypi.

The epithelium comes off easily in shreds. Ciliated epithelium are sometimes, even in old age, present in abundance. The cylindrical epithelium generally predominates.

Granular cells are present in abundance; free fat almost only in old persons; cholesterine I saw a few times.

There is much intracellular hyaline, and a few colloid globules. Blood corpuscles were in several cases very freely intermingled, and in one case only in a stellate contracted condition; less often I have found precipitated hæmatine.

The mucus secreted is generally whitish-gray, or reddish-gray, and either clear or clouded. In one case, I found it thin and fluid; in another, of the consistence of syrup; and, chiefly in those advanced in years, viscid. It is generally alkaline, although a variability from slight acidity to strong alkalinity has been noticed. The quantity found in the cavity may be as large as one drachm.

*Symptoms.*—Acute catarrh is, without exception, accompanied by dragging sensations in the lumbar and pubic region, which may radiate to the bladder and rectum. There is also often the feeling of a weight or pressure in the pelvis. The patient experiences similar pains when a chronic catarrh becomes exacerbated. The temperature of the body is always slightly, seldom much, increased; the pulse and respiration are quickened. The sleep is disturbed by frequent desires to urinate, and small, thin evacuations of the bowels, accompanied with rectal cramp, disturb especially the early hours of the morning. Most patients complain of slight chilly feelings, which alternate with flushes of heat. They speak quickly, are excitable or quick-tempered, and are inclined to be mournful. Some have headache, which is at first increased by their lying down, and they complain of megrim. They complain more of thirst than hunger, are often troubled with nausea, or pains in the stomach. They often declare that their abdomen is swollen, and cannot endure the bands of their clothes. In others, the lower portion of the abdomen is swollen, and at its centre (region of the uterus), or at the sides (region of the broad ligaments), is painful when pressure is made upon it. The skin is, as a rule, dry, and especially does the patient complain of a nocturnal burning of the soles of the feet; a few, also, of cold feet.

With severe lumbar pains, the sufferers frequently experience a discharge of clear mucus from the vulva; some have a discharge of bloody serum or of pure blood (*metritis hemorrhagica*). This discharge is seldom much in quantity, and lasts only a few days.

In chronic catarrh, the discharge is generally more considerable, but the local disturbances less. The fever is very slight. The gradual decline in strength is more marked; the face, previously of a good color, becomes pale, sallow, with dark rings around the eyes, and has the expression of pain. The patient grows thin, the menses are irregular, and constipation is often complained of.

Before puberty, catarrh of the body of the uterus, as far as I know, causes no annoyance.

### B.—IN CONNECTION WITH MENSTRUATION.

*Pathological Anatomy.*—The uterus is swollen and congested, its mucous membrane twice or three times thicker than normal, and on one wall sometimes thicker than upon the opposite one; its surface is covered over with slimy, partly coagulated blood. In rare cases, a large portion of the exuberant mucous membrane has been thrown off.

*Symptoms.*—Towards the end of the menstrual period, the beginning of which was ushered in by pains in the region of the uterus, and by an abundant discharge of mucus, clots of blood, together with shreds of mucous membrane, or a complete cast of the uterine cavity, are discharged, accompanied with bearing-down pains (*membranous dysmenorrhœa.*)

### II. CATARRH OF THE PREGNANT UTERUS.

Catarrh of the gravid uterus is of very rare occurrence, and an affection seen but by a few, for the reason that pregnancy is not favorable to the existence of catarrh of the uterine cavity, that its secretion is never discharged so as to be seen, and that *post-mortems* on pregnant women are not frequently available. I only know of one recorded case, and only in three cases have I myself been able to make examinations.

During the later period of pregnancy in many women, there flows from the vagina, in considerable quantity, a serous fluid, which, the speculum shows us, comes from the os uteri. The time of commencement and the manner of this flow are as variable as are its duration and its quantity. It comes on, for the most part, in the last week of pregnancy, less often in the last months. Krause,* from whom I borrow this assertion, adds: "Never before the seventh month." My case speaks for the possibility of a still earlier occurrence.

### HYDRORRHŒA OF THE GRAVID UTERUS.

While the woman is standing or walking, and even occasionally when she is lying quietly in bed, there occurs suddenly a discharge of fluid, without there having been any preceding pain. Only after a very abundant flow does there sometimes occur a pain somewhat like labor, just as if the uterus, having discharged some superfluous matter, contracts and closes intimately upon the embryo. Generally, this, after a short interval, recurs in a less violent degree, and continues to recur until labor begins; occasionally, after having been repeated a few times, it ceases, or a very slight discharge continues. The discharge is at first clear or yel-

* A. Krause, "Die Theorie und Praxis der Geburtshulfe." Berlin, 1853. II. Theil. S. 7.

lowish; later, mixed with blood, and has an animal smell. In the case of a strong, healthy primapara, I collected in the course of the fortnight preceding delivery, about four pounds of fluid.

The opinion that this discharge is the remnant of the hydroperion which is present during the first month of fœtal development, and which, having collected between the amnion and the vascular covering, is discharged through a rent in the chorion, is less probable than the hypothesis of Jorg, according to which this "false water," which in many cases is discharged a short time before parturition, originates from the allantois, which still exists as a cavity. The allantois, lying in the same space as the hydroperion, and continuing to exist as a bladder, gives rise to the fact that after birth the fœtal membranes seem, in the one case, a little, in another case much more, separated the one from the other.

Other and later authors, resting upon a few facts, have sought to deny *in toto* the occurrence of uterine hydrorrhœa. Ingleby, in 1832, reported a case of hydrorrhœa in which there was found a small opening in the fœtal membrane, close to the edge of the placenta, and totally unconnected with the large rent through which the fœtus had been born. Dubois observed a similar case. Danyan found the same. On the 12th of December, 1860, a woman was confined in the Maternite. She had during the five weeks preceding labor lost water almost continuously. Danyan discovered almost exactly opposite to the rent in the fœtal membranes a round opening three lines in diameter.

This condition, however, does not exclude the fact that the uterus, either when in its most congested state, and the decidua, when enduring so high a vascular pressure, do under favorable circumstances discharge on the exterior of the vascular membrane a portion of the serum serving the purpose of amniotic fluid; or become catarrhally affected, as Rhazes first demonstrated.

I learn with much satisfaction that A. Krause embraces my opinion. Suppose that the true fœtal water does originate for the most part from the deciduous membrane, the secretion of which, passing through the two fœtal tunics, arrives in the fœtal cavity; suppose, further, that the decidua induced by some cause, be it what it may, should quickly produce a greater quantity of water than the fœtal cavity is capable of containing, or that the decidua has at any point become separated from the chorion, then will the secretion, instead of passing through the fœtal coverings, be forced to accumulate between the vascular membrane and the deciduous membranes. The accumulating transudation follows the laws of gravity, and separates the union of the decidua and the chorion downwards until it reaches the os uteri, and is discharged in a gush. After the discharge, the passage is closed, whereupon the secretion accumulates at the original secreting point, until it again makes a way for itself outward. If, on the other hand, the passage remains open, or if the secreting point is situated quite near to the os internum, then the discharge does not occur in sudden gushes, but by drops. The occurrence of such an accumulation of the fœtal fluid, destined for the fœtal cavity, between the uterus and the fruit, is also favored by the fact that, if exhaustion be present, this transudation decreases, whereas where there is extraordinary energy there more is produced than is necessary. In this category belong also the cases* in which œdema of the uterus was the source of the hydrorrhœa. To refer them always, however, to uterine œdema (Hohl) would be wrong.

With Krause, I consider it not impossible that this fluid is occasionally the product of the mucous membrane in the course of development beneath the decidua, from which mucous membrane the old decidua separates itself the more as the end of pregnancy approaches, consequently in such cases the transudation is serous mucus. Rigby was of the same opinion.

C. Braun was the first who examined microscopically the locality of the increased transudation. He found the region of the decidua scrotina, hence the external superior surface of the placenta, covered with a new growth rich in cells, which caused him to deduce the existence of inflammatory irritation. The increased exosmosis is further spoken of by J. Harrison.† He found in every case a fibrous band at the border of the placenta, and refers the water accumulation to the space between the fœtal membranes. C. Braun gives further confirmation when he asserted that chronic uterine catarrh is not relieved by pregnancy, and also P. Dubois and Chailly, who found in some women the hydrorrhœa still continuing after parturition.

Case I.—From my private practice. Mrs. E., in Leipsic, 32 years old, was delivered of her first child by means of the forceps. This otherwise healthy woman was attacked repeatedly with intermittent fever, which was here endemic, and bore in pretty rapid succession three more children; was, however, during the last pregnancy considerably œdematous. The vaginal portion of the uterus was also a good deal tumefied.

On the 31st of July, 1859, her menstruation ceased. During the end of December occurred a mucous discharge from the genitals. In the course of the following days blood was added to the mucus. Without any further cause, hydrorrhœa occurred on

* L. F. Wokaz, "Diss. Exhibens Graviditatis et Hydropis Uteri, Ambiguæ Exempla." Lips., 1813.—J. B. Geil (præs. F. C. Naegele), "Diss. de Hydrorrh. Uteri Gravidarum." Heidelb. 1822.—Paffermann, Lips., 1835.

† British Medical Journal, June 27, 1857.

the 7th of January, 1860, at 7½ P. M. The discharge was also streaked with blood. At 9¾ P. M., of the same evening, the midwife removed from the vagina the fœtal mass, the membranes being unbroken. Attached to the placenta was a club-shaped fleshy mass; its exterior consisted of young cells, its interior of crude blood coagula. The chorion was raised up as much as three millimetres by the exudation from the internal surface of placenta. At some points the placenta was paler and harder to the touch. At other points were embedded fresh apoplexies arising on the uterine side of the placenta.*

After the puerperal state the woman suffered repeatedly from leucorrhœa, accompanied with an enlarged uterus, which was tender to the touch, and occasionally visited with drawing pains.

In July, 1860, she again conceived. On the 9th of November she had chilly feelings along the back; in the evening, about nine o'clock, occurred all of a sudden a flow of waters, after that for a long time only thin mucus. On the 8th of December she bore a healthy female child, which she nursed for three weeks. Six weeks after her confinement menstruation returned.

CASE II.—Under the care of Professor Crede. A public woman of delicate build, twenty-three years old, was treated here in the hospital until the end of November, 1861, for a syphilitic ulcer of the vulva, and was transferred to the Trier's Institute on account of premature labor in the eighth month coming on. The patient in the few previous days had lost a great deal of clear water, which later became more scanty and bloody. The child was born alive. On the after-birth were seen the membranes, which had only one large rent, but they were discolored, especially on the placenta; the umbilical vesicle, half an inch from the edge of the placenta, was shriveled up between the membranes. On the other side of the decidua, which was drawn together into a thick ridge prominent upon the edge of the placenta, a gelatinous mass ran round the third part of the circumference upon the external edge of the placenta about three lines broad, two lines thick, of a dull yellowish-gray, here and there colored with blood, which could easily be separated in the direction of its long fibres, but could not very easily be torn transversely. This mass showed under the microscope spotted striæ and fusiform bands. With both these kinds of plasma membrane were associated cells, the more prominent of which contained each an elongated, umbilicated nucleus; at one point there were numerous, round, very delicate cells, having a diameter of 0.0057 to 0.0133 millimetres, with hyaline contents,

* C. Hennig, "Ueber die Nebenbander und Schafhautstrange in der Eihole des Menschen, *Archiv. fur Pathol. Anat.*, Bd. xix., Hft. 1., S. 200.

and each having from one to three nuclei with distinct outlines, which, upon the addition of acetic acid, became still more distinct, while the plasma membranes cleared up until they disappeared. The nuclei, which in many cases were in the act of dividing, had diameters of 0.0044 to 0.009 millimetres, and contained each one to two nucleoli. I looked upon these cells as the source of hydrometra.

Case III.—From the clinic of Professor Germann. Mrs. Gressler, of Weimar, forty-three years old, had had very painful menstruations, had had eleven children, four miscarriages, the last in September of the previous year. During the present pregnancy, which had already reached the end of the seventh month, she experienced constant pain in the lumbar region, had no appetite, and the stools were hard and infrequent. In the beginning and middle of October she had considerable watery discharges from the vulva.

Not only in this case, but also in another case of Germann's obstetrical clinique, did hydrorrhœa occur before the seventh lunar month. On the 24th of November labor-like pains occurred, and likewise symptoms of metro-peritonitis. Dover's powder and warm poultices to the abdomen were ordered. Death occurred on the evening of the 27th.

During life hard lumps could be felt posterior to vaginal portion of the uterus, extending as far up as the promontory. They proved to be cancerous lymph ganglia. The uterus presented to the naked eye only upon its posterior surface yellowish-white medullary-cancerous masses, here and there covered by fibrinous exudation. They extended from the ligamenta ovarii outward along the oviducts, which otherwise were not strikingly affected, upon the peritoneum uteri. While the cavity of the fœtal membranes contained greenish-brown, turbulent, amniotic fluid, there was found, in the space between the chorion and the decidua, which latter was completely separated from the former, about a tablespoonful of bloody serum, the remainder having flowed away during the discharge.

In this transudation were found, besides blood-corpuscles, which had partially lost their color, cylindrical epithelium, which were 0.076 to 0.095 millimetres long, 0.01 to 0.015 millimetres broad, with one to two round or oval large nuclei. More abundant were round, or elongated, or fusiform cells, having from one to eight large nuclei; some of them changing into hyaline. These cells also, according to the assertion of E. Wagner, to whom I showed the specimen, were not distinguishable from cancer cells. There were also present large round or egg-shaped bodies without nuclei, and in which no nuclei could be made manifest upon the addition of acetic acid. And, finally, there were long, flat fibres, many of them fusiform, evanescent upon the addition of acetic acid. The decidua was composed of fibres, like those first described, which were plentifully permeated by pearly, flat globules, arranged side by side in rows; and of large, nucleated, irregular, and many-tailed cells, whose nuclei were blood-stained. They resembled in shape granular cells and flat epithelium.

### III. CATARRH OF THE PUERPERAL UTERUS.

During the puerperal state, catarrh of the uterine cavity is rare. On many occasions, and, in particular, in institutions where putrefying animal matters are present, and communicated to the pregnant and lying-in women by those present administering obstetrical aid—even when cleanliness of the vagina has been neglected—it becomes developed in a surprising rapidity and frequency, and is communicable as well by contact as by means of the atmosphere.

Thus uterine catarrh is, then, the common cause and true seed of puerperal fever. Instead of the bloody lochia, there is secreted, for several days, almost nothing, or an almost colorless fluid, and then only, with the increasing endometritis, is occasionally bloody or ichorous matter discharged.

*Complications of Catarrh of the Cavity of the Body of the Uterus.*—The lengthening of the uterine cavity depends more upon the accompanying hypertrophy of the organ than upon a dilatation of the cavity, which, except in cases of hydrometra, with occlusion of the os, can never, on account of the dependent position of the canal, obtain to any extent. West found, in an inflamed and flaccid uterus, over one drachm of pus. Whether catarrh of the cavity can cause a considerable hypertrophy is, on account of the difficulty of the diagnosis during life, still uncertain. We find ourselves here, in many respects, in the same embarrassment as when speaking of the concomitant phenomena of tubal catarrh; and I have, through tedious researches, hardly advanced further than Ch. West,* when he says: "Our means of examining the condition of the womb are very imperfect, compared with those that we possess for investigating the state of other organs; and hence the question often arises, whether the signs of disease which we discover are the cause of the symptoms, or whether they are the index of other and more important changes, or whether they are neither the one nor the other, but mere casual concomitants of graver ailments, concerning whose nature and degree we can, from them, deduce no conclusion."

* Ch. West, "Lectures on the Diseases of Women." Lond., 1856. Part I, page 99.

ETIOLOGY.

In the case of the uterus we can more directly conclude whether, and in what way, its mucous membrane is affected by irritants, because it is more openly situated to the outer world, and is more frequently primarily diseased than the oviducts.

I. *Idiopathic Catarrh.*—Mechanical injuries comparatively seldom affect the non-pregnant uterus, on account of its small size, but as the result of severe injuries, there occurs more frequently hyperæmia, hemorrhage, or inflammation of the serous covering, together with secondary catarrh, than primary and simple catarrh. Still, there are several cases to be found in literature in which mechanical injuries have been followed by primary catarrh. Severe shocks, either physical or mental, are especially injurious during the child-bearing period. If there be therewith great or long-continued excitement of the circulation, as in immoderate dancing, wine-drinking, anger, then there is sufficient cause for the production of uterine leucorrhœa.

Aran told me personally that in Paris he had come to the conclusion that uterine catarrh arises in the rich classes from emotional excitements, especially depressing influences, but, in the poor classes of the population, from tuberculosis of the lungs. I can in this place, as far as my experience goes, confirm this assertion. Violent coition is more injurious than too frequent; often still more so is an unnatural and unsatisfied excitement. Incongruity between the genitals of husband and wife, and the resulting unhappy wedlock, may possibly outrank the previously cited cause.

*Physico-Chemical and Atmospherical Irritants.*—Too cold, too frequently repeated, and too strong injections more often cause uterine catarrh than does the unskilled sounding of the uterine cavity, as the latter causes rather wounds, hemorrhages or uterine pain—neuralgia or cramp. Not unfrequently styptic agents which have been used to check uterine hemorrhage act as additional irritants. Too warm clothing, too soft a bed, sitting over furnaces, are in part directly, in part through the effeminateness which they cause, injurious, inasmuch as the liability of catching cold is thereby increased. Too sudden changes of temperature are, to susceptible persons, the more injurious in proportion to the highly-excited condition of the uterus, and in proportion to the proximity of the cold-causing or heat-abstracting object to that organ: as sitting upon a cold stone after a fatiguing walk, standing in the moist grass, in a cellar, the going barefoot of such persons as are not used to it. Many women, who otherwise dress themselves very judiciously, have the unreasonable habit of going out in rainy and snowy weather in the thinnest of shoes and stockings; others have the still more pernicious habit, when bathing in a river or the sea, of going slowly into the water, instead of either first pouring cold water over themselves, or, at least, of bathing the upper portion of the body, and then immediately diving into or running quickly into the water.

Of poisons, the ethereal oils, the strongly bitter and balsamic agents (sabina, turpentine oil, cantharidin, lupulin, copaiba), alkalies, acids, and several neutral salts, have long been in ill-repute. It is well known that some of them—nicotianin,* sulphuric acid,† have been detected in the secretion of the endometrion; of others, it is supposed, and upon good ground, and deduced from the collected experiences in the case of the urinary organs and the rectum, that they act as irritants to that mucous membrane through which they are excreted from the blood.‡ Daily observation shows that others change the character of uterine discharges (cubebin), sometimes curatively, or intensify acute discharges and aggravate chronic ones (sulphates of the alkalies, iodide of potassium, muriate of ammonia).

During pregnancy, infection with blennorrhœal secretion, as a result of connection, may act injuriously; in childbed, the remaining behind of a portion of the placenta or of the membrane, and the endeavors to remove these, as also the operations which, during parturition, may have become necessary, tilting over of the uterus, the poison of a cadaver, or of a disease which may be introduced by the finger of the examiner, exposure to cold, mental emotions, badly-ventilated rooms, damp atmosphere, and drastic medicines.

II. *Catarrhus Deuteropathicus.*—1. *Symptomatic.* (*a*) Flexures and curvatures of the uterus are decidedly more injurious than are changes in its position, for by changes in its form there is caused a more or less stasis in the blood and lymph-vessels, and, in an aggravated case, the excretion will be retained in the uterine cavity.

Among my twenty-eight cases of catarrh of the uterine cavity were three cases of flexure, and once the body of the uterus was swollen so as to be a case of hydrometra.

(*b*.) The uterine cavity I found reduced in size several times through hypertrophy of the connective tissue and of the muscular layer, once through an adhesion of the opposite mucous surfaces in the right horn of the uterus, once through a submucous fibroid; in old persons, often through ecchymoses, cysts, polypi, and tufts.

* Melier, *Bull. de l'Acad. de Med.*, x. p. 585; and Wertheim, *Zeitschrift der Ges. der Aerze zu Wien*, Jan. 1851.

† *Med. Gazette*, May, 1828.

‡ C. Hennig, "Chemische und pharmakologische Prufung des Gummi Kino," *Archiv. der Pharmacie*, cxxiii., Bd. 2, S. 158; und *Arch. fur Physiol. Heilkunde*, xii., S. 612.

Concerning the constrictions and occlusions of the internal os we shall speak more particularly later.* At present we will merely state that in three cases dropsy of the uterine cavity was found.

2. *Sympathetic.* It is perfectly obvious, from the greater frequency of tubal catarrh over catarrh of the uterine cavity, that tubal catarrh is more often produced by uterine catarrh than is uterine catarrh by leucorrhœa tubaria; as an anatomical proof, we mention the difficulty with which viscid mucus passes through the oviducts into the uterus through that very narrow portion of the tube which is situated in the substance of the uterus. Furthermore, it must be borne in mind, that the cause of irritation for the mucous membrane is more surely transmitted from tissue to tissue than through the medium of a mucous vehicle.

3. Catarrh is a result of congestions and hyperplasmas (muscular hypertrophy, fatty degeneration), and of heterologous affections of the uterus, whether it be cancer, tubercle, cystosarcoma, or a dermoid tumor.

While I admit that gonorrhœal catarrh of the uterus frequently produces a mucous secretion in the oviducts, yet I venture to doubt that that tubal mucous secretion is infectious; otherwise it would, by its very pertinacity, through the continual current uterineward kept up by the ciliary movement, re-excite gonorrhœal catarrh in the already cured uterus. The analogy is also wanting in the case of men patients. How very often there remains, after the disappearance of the urethral gonorrhœa, a swollen condition of the prostate gland, catarrh of the bladder, or a tendency to inflammation of the epididymis, without there being caused, through a renewing of the inflammation in the last-mentioned part, or even through a rekindling of a mucous discharge from the bladder, a second renewal of the affection in the urethra.

As regards gonorrhœa of the uterus, it is in any given case difficult to say whether it is primary, that is, caused by immediate reception of the blennorrhagic mucus from the urethra of the male, or has occurred secondarily, that is, communicated from the vagina to the cervix uteri, and so on further up. It is also supposable that frequently simple catarrh of the uterus or phlegmorrhœa of the vaginal portion accompanies vaginal gonorrhœa.

Inflammation and foreign growths of the tubes, of the ovaries, or of the uterine ligaments (peritonitis), frequently also cystitis, are accompanied by a uterine mucous discharge. Still more intense than those are the effects in individual cases of peritonitis and severe intestinal catarrh, cholera, and enteritis, together with diarrhœa. Here may be mentioned also the pelvic abscesses so frequent as compared to the very seldom occurring abscesses of

* Compare "E. Wagner, der Gebarmutterkrebs." Leipzig, 1858. S. 44.

the uterine substance, and whose precursor and companion uterine catarrh may be.

Uterine catarrh occurs secondarily from rigidity and occlusion of the uterine arteries, from capillary embolus, and from dilatation of the veins; the latter frequently the result of overdistension and relaxation of the veins of the pelvis in general and of the hemorrhoidal veins in particular. The walls of the veins are thereby rendered thin, their power of contraction diminished, and they are occasionally in a state of fatty degeneration. This condition is produced by an improper use of burnt coffee, in a less degree of tea, by an excessive use of sleep-inducing drinks and warm soups, by long-continued constipation of the bowels or by the misuse of powerful cathartics, or follows as a result of frequent and difficult labors. It often leads to thrombosis of the hypogastric and uterine veins.

According to my experience, such conditions are also caused by abnormal depositions of fat in the periuterine cellular tissue. They also accompany unalterably most cases of heart disease, severe cases of emphysema, tuberculosis, frequently, also, Bright's disease, and serious diseases of the liver and spleen. Consumption is also the cause of uterine catarrh in those cases in which the pressure of the blood in the pelvic vessels is increased; it is, nevertheless, worthy of notice that tubal catarrh is more often found in connection with tuberculosis of the lungs, than is uterine catarrh. To what extent the scrofulous diathesis favors the occurrence of uterine catarrh, I am not in a position to say.

I am firmly convinced that chlorosis, hydræmia, typhus, and the acute exanthemata, give rise to and maintain it.

During childbed, uterine catarrh is so rare that in the lying-in asylums of this city I have never seen it, except during puerperal fever. Among the poorer classes, where the mother often rises upon the first or second day after parturition and scrubs her room and makes her coffee, it occasionally occurs. Puerperal tubal catarrh forms to this a striking contrast, although it is also comparatively rare.

### CONSEQUENCES AND RESULTS.

I am authorized in the opinion that uterine catarrh gets well easier than tubal catarrh. Thickening, roughness, and inflammation of the peritoneum of the uterus occur often enough simultaneously with catarrh of its cavity, or with traces of it, not to excite attention. Granted that catarrh is often a result of metroperitonitis, we have still, also, cases of old catarrh and recent peritonitis, and besides that the fact that primary inflammation of a serous membrane is rare, especially in the case of the peritoneum. Now, we meet with in the catarrhal uterus only occasionally hyperæmia or inflammation of the parenchyma; therefore the peritoneal irritation originates from either an acute catarrhal inflammation which has been associated with uterine hyperæmia, or from an irritation of the mucous membrane, which was simultaneous with a physiological uterine hyperæmia, namely, menstruation or pregnancy.

And, in fact, there are complained of, especially at the time menstruation begins or is about to occur, and likewise frequently in the first part of the last months of pregnancy, pains which point to a stretching of the inflamed serous covering combined with meteorism and constipation, and to a rupturing of the already formed adhesions and bands, as we almost daily in leucorrhœal women can conceive of, even feel and after their death find. Too often these dragging pains are considered labor pains or nervous colics.

And should this mode of propagation of the irritation be the exceptional one, there is no difficulty in coinciding with the belief of celebrated authorities, that catarrhal excitement in the uterine cavity is followed by corresponding participation on the part of the tube, and its fimbriated extremity is the point in the human body where mucous membrane and serous membrane, without anything intervening, border the one upon the other. This so dangerous approximation only occurs on one other occasion, namely, at the time when the ruptured Graafian follicle of the ovary has not yet closed again.

The oviducts suffer, therefore, most severely when the catarrhally swollen or even irritated mucous membrane of the uterus closes up their uterine openings. In this connection may be considered—

DROPSY OF THE UTERINE CAVITY (ACCORDING TO RHAZES).

It is relative in its character when the internal os is only occasionally constricted, or is occluded in such a manner that under favorable circumstances it again becomes pervious, as for instance, through spasm of its circular fibres, through acute swelling, or a casual forelying polypus of the mucous membrane, or through flexure. Otherwise it is absolute. Only in one of these three cases was the right tube impassable; therefore the contents of the uterine body could have discharged itself through the left tube, if this procedure were so easy of accomplishment; in the other cases the interstitial portions of the tubal canals at least were constricted.

In two cases the contents of the hydrometra had a slightly acid reaction; in one it was gelatinous and transparent, in the other serous and opaque. In the third case the very abundant mucus was alkaline, and contained great numbers of epithelium from the glands and cells from the surface of the mucous membrane.

The uterus is more often hypertrophied than by pressure of the fluid atrophied. We shall see later that highly developed Nabothian bodies in the cervical canal also obstruct the discharge of the uterine mucus.

The muscular tissue of the uterus from oft-repeated hyperæmia and from serous infiltration, both of which conditions in a more or less degree attend catarrh, becomes softer and weaker, or from repeated endeavors to rid itself of the contents of the cavity of the body, hypertrophied. A mixed condition is brought about by hyperplasia of the connective tissue, which is known as "chronic infarction."

The mucous membrane during chronic catarrh undergoes very appreciable changes. It becomes permeated by varicose vessels, and scattered through it are ecchymoses and deposits of pigment; it loses its ciliated epithelium, or the ciliæ do not move regularly. The tubular glands become producers of fat and hyaline instead of healthy epithelium, and finally many of them, their emunctories being oculated, become changed into cysts, several of which, the one alongside of the other, may form into a common cone which may become detached. They may form "cellular" or "vesicular" polypi with broad or pediculated basis.

In all cases, the general health of the patient suffers; the frequent pains and discharges, but especially the sleepless nights and fatty digestion, reduce even the stronger patients.—*American Journal Obstetrics, etc.*, May, 1872.

